# A Protocol for the Ethical Assessment of Wild Animal–Visitor Interactions (AVIP) Evaluating Animal Welfare, Education, and Conservation Outcomes

**DOI:** 10.3390/ani9080487

**Published:** 2019-07-25

**Authors:** Barbara de Mori, Linda Ferrante, Daniela Florio, Elisabetta Macchi, Ilaria Pollastri, Simona Normando

**Affiliations:** 1 Department of Comparative Biomedicine and Food Science, Università degli Studi di Padova, viale dell’Università 16, Agripolis, 35020 Legnaro PD, Italy; 2 Ethics Laboratory for Veterinary Medicine, Conservation, and Animal Welfare, Università degli Studi di Padova, viale dell’Università 16, Agripolis, 35020 Legnaro PD, Italy; 3 Department of Veterinary Medical Science, Università degli Studi di Bologna, via Tolara di Sopra 50, 40064 Ozzano dell’Emilia BO, Italy; 4 Department of Veterinary Science, Università degli Studi di Torino, largo Paolo Braccini 2, 10095 Grugliasco TO, Italy

**Keywords:** animal–visitor interactions, ethics, animal welfare, zoo visitors, risk assessment, *Giraffa camelopardalis*, ethical matrix, wildlife tourism, conservation education, One Welfare

## Abstract

**Simple Summary:**

Animal–visitor interactions are the experiences offered by zoos, sanctuaries, and other tourism facilities in which people can be very close, and even touch, wildlife. This proximity could damage animal welfare and be a risk for the health of both animals and visitors. Proximity, however, has a positive emotional impact on visitors, representing an excellent opportunity to communicate conservation and educational messages. We present a protocol to evaluate interaction activities, and describe its application in a “giraffe feeding” interaction evaluation. Behavioral observations and a risk assessment evaluated the impact on animals. A risk assessment related to both visitors and staff and a questionnaire investigated the risks for people and the emotional, educational, and conservation outcomes. An ethical analysis, using an ethical matrix and a checklist, integrated the results, and identified the possible ethical concerns of the interaction. Giraffes’ behavioral freedom and welfare were safeguarded, and a positive emotional and conservation oriented impact was found, the only improvement that could be suggested, in case of restructuring of the facility, being the absence of hand washing facilities after the interaction. The protocol showed its potentiality to protect animal welfare and human health and to promote an ethical use of the interactions.

**Abstract:**

Due to the popularity of wild animal–visitor interactions (AVIs), there is a need for an ethical assessment of their impact on animal welfare, education, and conservation. The protocol presented in this study is designed to evaluate such interactions on an integrated level, using a transparent analysis of all the aspects involved, including all the stakeholders and the potential conflicts of values. The protocol consists of a six-step process encompassing dedicated data acquisition and a specific ethical assessment. When the protocol was applied to assess a “giraffe feeding” interaction, steps devoted to data acquisition found that animal welfare risks were low, and that visitors described giraffes with emotionally linked descriptors more often after the interaction. The net promoter score, which refers to how likely visitors would recommend to a friend to join the animal–visitor interaction, was 74%. The subsequent ethical assessment, which consisted of a comparison of the results of the previous steps with an ethical matrix highlighting the ideal situation for all stakeholders’ interests, allowed the overall identification of the ethical concerns entailed by the interaction. A final ethical checklist of the examined AVI had a “yes” in entries regarding animal welfare, emotional, and conservation mindedness outcomes and ethical assessment.

## 1. Introduction

Facilities that home wildlife animals, such as zoos and aquariums, as well as sanctuaries, rescue centers, and game reserves, often offer interactive experiences to their visitors. Seventy-five percent of zoos and aquaria (i.e., 929) included in a recent review [1] advertised at least one form of interactive experience with the animals on their webpage. This practice, apart from being entertaining, is expected to promote education efficacy [2,3,4], with education being a key objective for the modern zoos and similar facilities [5,6,7]. Although animal–visitor interactions (AVIs) are popular, the animal welfare, educational, and ethical impact of such interactions is still relatively unknown, and more research is required [8,9].

The Animal Welfare Strategy “Caring for Wildlife” and the Guidelines for “The Use of Animals in Visitor Interactions” of the World Association of Zoos and Aquariums (WAZA) [7,8] recommend the adoption of a policy to ensure that animal welfare is guaranteed at all times during AVIs. Furthermore, WAZA guidelines state that “responsibilities include considering the safety of the public and the animals, regular evaluation of the relevance of the interactive experience and the ability of the message being delivered to encourage subsequent responsible behaviours” [8] (page 2). A regular assessment of all interactive experiences is strongly advised in order to check their impact. Animal-focused evaluations should be used to estimate the physical and psychological state of the animals involved, and of all other individuals in the social group or exhibit. Indications are also provided for the suitability of the species, the required staff expertise, educational messaging, safety, and monitoring and assessment. Additionally, ‘appropriate’ ethical reviews on the suitability and impact of the interactive experiences” [8] (page 3) are recommended. The assessment process suggested by WAZA’s Guidelines and Welfare Strategy can help zoos, aquariums, and facilities offering interactive experiences, to state and communicate the ethical principles and values that guide them. By applying a review assessment process and striving for continuous improvement, facilities can demonstrate their commitment to promote animal welfare and educational standards [10].

To fulfill their goals concerning both animal welfare and conservation education, and to increase their reputation among the public, zoos and facilities alike need to apply transparent procedures to evaluate their programs of animal–visitor interactions at an integrated level and to be prepared to discontinue interactions that have an obvious detrimental impact on the animal’s welfare.

WAZA’s Guidelines and Welfare Strategy need to be applied in daily management. The aim of this paper is to describe a protocol designed to apply WAZA recommendations. This protocol should be adaptable to different types of experiences in zoos and other facilities (feeding interaction, walk-with and pet, touch pool, walk-through enclosure, etc.), and to different management approaches (pre-booked experience or free activities for all visitors). A significant number of different activities involve animals in interactions with visitors, from riding to feeding or just walking inside the exhibit and a significant number of different species, ranging from giraffes to reptiles, are involved in animal interactions. Maintaining a high standard of welfare for each animal—before, during, and after each interaction—is difficult due to the diversity of animals involved. Another challenge with animal interactions is not only the different species but also that different individuals will vary in their response to these experiences.

In Section 2, it will be presented the general architecture of the protocol to be adapted to the many different situations in which AVIs can take place. In Section 3, Section 4, Section 5 and Section 6, an example of its application will be given, using the case of a visitor feeding interaction with giraffes.

In the “giraffe feeding” AVI, the assessment based on the protocol presented here found low animal welfare risks, but respondents described giraffes with emotionally linked descriptors more often after the AVI. People showed an appreciation of the AVI, the net promoter score being 74%. The final overall assessment was based on a checklist in which the examined AVI had a “yes” in entries regarding animal welfare, emotional and conservation mindedness outcomes, and ethical assessment (entries 1, 3, 4, 7, 9, 10, 11).

The protocol showed promise in investigating the different facets of the AVI experience and to identify the related ethical concerns.

## 2. The Animal–Visitor Interaction Protocol (AVIP)

The Animal–Visitor Interaction Protocol (AVIP) presented here may allow AVI offering facilities to monitor and improve their interactions. AVIs here are intended as any activity that allow close proximity between visitors and wildlife animals, where direct contact with the animals is allowed (touch pool, animal feeding, animal riding, etc.) or where visitors enter the animal exhibit (walk-through, swim-through, etc.). Open training sessions or interpretative talks where visitors do not interact directly with the animals are excluded. Similarly, in this study, we do not consider situations in which visitors can be admitted inside safari park exhibits with their cars or into bird aviaries by feet.

AVIs are often the focus of public debate because, on one hand, they may create educational opportunities [11], whilst on the other hand if not correctly planned and monitored, they can have negative outcomes on welfare and health for both humans and animals [12]. Interactions are not bad or good by definition, but they must be assessed to confirm their value.

The AVIP is designed to assess and monitor AVIs, as it helps to identify the strengths and weaknesses of the interaction and supports the staff in highlighting specific problems and finding acceptable solutions. Furthermore, AVIP may help to promote animal welfare assessment by using a uniform protocol in zoological facilities as advised in the Animal Welfare Strategy of WAZA [7]. Moreover, AVIP helps to promote the idea that not only zoological facilities and aquariums, but all the facilities offering wildlife AVIs should assure the same high standards of safety and welfare to animals, workers, and visitors. Independently from the facility’s mission (e.g., wildlife conservation, wildlife tourism, maintaining injured animals not able to be released into the wild anymore, etc.), acceptable standards of health safety and animal welfare must be granted and a take-home educational message should be provided if wildlife encounters are offered. The general aims of the AVIP are to promote awareness of the pros and cons of animal interactions and to increase the quality of these experiences.

To increase their quality, AVIs, besides the initial assessment, should be monitored regularly in order to identify a possible decrease in the standards. The changes may be due to several reasons: Modification in the animal management, differences in the animal group’s dynamics, shifts in the visitor target, etc. The periodic application of the AVIP meets the need for monitoring and documenting the assessment, as requested by WAZA, and allows to compare the effects of different management strategies.

Considering the complexity of AVIs, only the implementation of a multi-disciplinary approach can help professionals in the assessment of these activities. A standardized assessment, based on a protocol which can be applied in a uniform way, can help zoo staff in their choice of suitable species and/or individuals, the adoption of adequate evidence-based animal management practices, the planning of convenient exhibit design for interactions, and the selection of a responsible take-home message for visitors. The use of a uniform protocol, such as the AVIP, can help to improve the overall ethical approach and consistency in management decisions. Moreover, the dissemination of the results of the AVIP can be an opportunity to communicate the animal welfare and wildlife conservation commitments of the facility to visitors and other stakeholders, improving transparency and thus increasing the facility’s reputation [13].

**Scheme 1:**
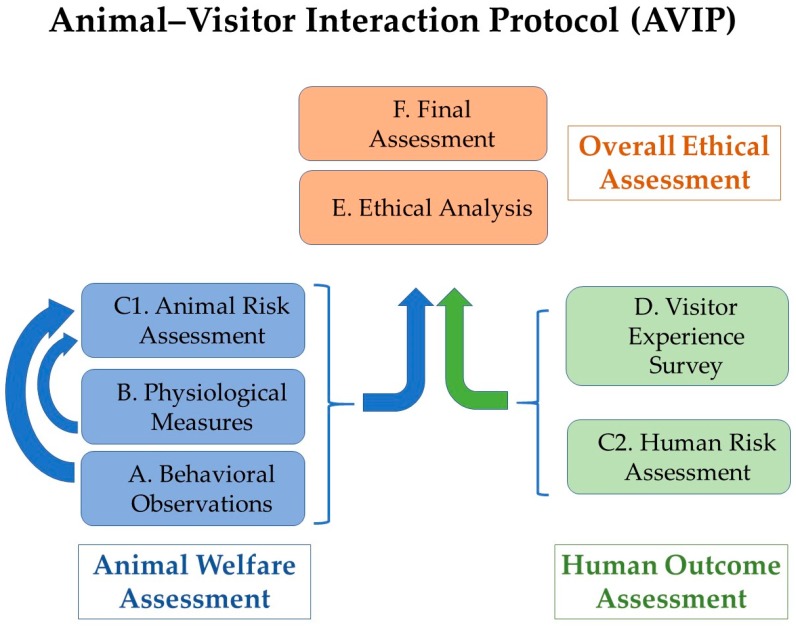
The diagram of the animal–visitor interaction protocol (AVIP). The six steps of the protocol (steps A–F) are shown with their relative connections. Step C is divided into two parts: C1 focuses on animal risk assessment, whereas C2 focuses on risk assessment related to staff and visitors. The steps represented by blue boxes are the ones dedicated to animal welfare assessment. Results of step A and B are the base of step C1. Steps C2 and D are represented by green boxes and focus on human outcome assessment. Step E and F are represented by orange boxes and correspond to the final phase of the protocol being the overall ethical assessment part of the protocol.

The suggested protocol (Scheme 1) was designed to assess each AVI considering its different facets, by grouping them into six steps and three “assessment areas” (Animal Welfare, Human Outcome, and Overall Ethical assessments):

- Behavioral observations and analyses;
- Physiological measures;
- Risk assessment;
- Visitor experience assessment;
- Ethical analysis;
- Final assessment.

### 2.1. Animal Welfare Assessment

The first assessment area of the protocol consists of a multi-facetted approach to evaluate animal welfare during the interaction (for more details about steps from A to C1, see Normando et al. [14] where the application of this assessment area to the AVI example of “giraffe feeding” is discussed. Some suggestions on this approach are provided in the following).

In step A, comparison of the behavior expressed by the animals during (and around) the presentation of the specific stimulus (i.e., the interaction) with that expressed when the stimulus is not present, appears as most adequate, because the AVIP aims to assess the possible animal welfare risks that are specifically related to a relatively short-time event and not to the general housing/management conditions. Of course, potential confounding factors should be controlled as much as possible. An important factor when interpreting welfare related behavioral results is whether the animal has freedom of choice, because control over the environment (in this case being able to choose whether to interact or withdraw) is important for stress perception [15,16,17,18].

Step B is focused on physiological parameters, and using such physiological parameters together with behavioral ones when assessing welfare, is a procedure that is useful and thus is usually recommended in order to have a more reliable interpretation of the results [15].

The application of risk assessment procedures in step C1 is a relatively novel approach to animal welfare for wild animals in human care, even more so when the effects of AVIs are concerned. Since there is a lack of specific knowledge about how uncertainty can affect the evaluation (due to the multidimensional nature of the concept of welfare) the AVIP suggests to carry out a semi-quantitative assessment in which exposure factors and welfare consequences are expressed at first in qualitative terms [19]. They can then be transformed into numbers to be processed in the dedicated calculation algorithms in the risk characterization phase. C1 suggests also a checklist investigating how adequate the enclosure is for maintaining a high standard of welfare during interactive activities and how suitable management is in preventing risks for the welfare of the animals.

### 2.2. Human Outcome Assessment

The second assessment area concerns the evaluation of the outcomes for the people involved, i.e., visitors, keepers, veterinarians, and other staff. Outcomes include both benefits and harms (i.e., risks and actual damages), and span several domains: Welfare (including physical health and emotional states and feelings), education and conservation mindedness, thus recognizing the link between animal welfare, human wellbeing, biodiversity, and the environment, as recently encompassed in the “One Welfare” concept [20]. Topics concerning education should cover both conservation and animal welfare as suggested by the WAZA Animal Welfare Strategy [7] and the EAZA (European Association of Zoos and Aquariums) Conservation Education Standards [21].

The most intuitive part of the human outcome assessment is the evaluation of health risks (i.e., those caused by infectious/parasitic agents, allergies, and possible traumas) for both visitors and staff involved in the interactions, as illnesses and injuries are highly likely to have a negative impact on the welfare of the affected individual [22]. However, illness and traumas are not the only issues that can negatively impact on the welfare of the people involved in AVIs. Other potential hazards can result from a negative emotional impact of activity with animals (e.g., Frommer and Arluke [23]) such as moral distress and burn out [24] or compassion fatigue [25]. Furthermore, visitors can be negatively emotionally affected by the AVIs, if for example, the experience does not meet their expectations in terms of emotional enjoyment, knowledge gained, kindness of the staff, possibility to do what they wish or, for some people, if they perceive that the animals are not well treated.

Concerning human health in step C2, AVIP follows DEFRA (the Department for Environment, Food and Rural Affairs) [26] and EAZA [27] documents, which are oriented to identify the necessary measures to eliminate or reduce hazardous exposure and identify preventive and protective measures that, directly or indirectly, guarantee the protection of people. For physical or biological risks, a risk assessment procedure that encompasses the standard phases of identifying hazards and characterization, exposure assessment, risk characterization, and post preventive and protective actions implementation control is included in the protocol.

The assessment of the other aspects of human welfare (i.e., the ones relative to emotions, feeling, education, and conservation mindedness) that can be impacted upon by an AVI, have to be addressed separately for visitors and staff. A visitor experience survey (step D) can be used for visitors and an ad hoc structured interview can be used for the staff members directly involved in the AVI, in case the responses of the staff to informal engagement with the researcher, high staff turnover, and high risks for injuries linked to AVI performance, suggests there could be problems in this area.

Step D investigates changes in visitor attitude towards animals and conservation issues together with their perception of the experience and their motivation and expectations around the interactions. 

Visitors’ characteristics such as age, level of education, degree of awareness on conservation issues, and eventual misconceptions can heavily influence visitor understanding and involvement, and consequently, affect the final educational outcomes of AVIs. Therefore, a front–end evaluation on visitors could be an asset before launching a new AVI to better focus the target audience. In general, a bottom–up approach shows to be a suitable way to increase inclusiveness and effectiveness in education programs in zoos and aquariums [28].

The use of a preliminary visitor analysis checklist, recording information given to visitors, is advisable in order to tailor step D survey questions to the actual interaction being investigated. It is also useful to design different versions of the questionnaire to be administered to different kinds of visitors (participating or not participating in AVIs) and at different times (before and after the interaction). Comparing data before and after the interaction, together with data gathered independently from the AVI, can help in understanding what the outcomes of the interaction are and which outcomes are linked to the interactive experiences and which ones are due to the overall visit of the facility.

Moreover, an analysis of the answers given to the questionnaires is useful to understand visitors’ points of view and ethical values, thus contributing to filling in the matrix for the ethical analysis (step E).

### 2.3. Final Overall Ethical Assessment

The third and last assessment area concerns the different ethical issues that AVIs may pose, such as the potential negative impact on animal welfare, and the possible conflict of interests among the animals and the management of the zoo and the visitors [29]. Step E has the main goal to make these issues explicit by means of a systematic consideration of the needs and values of the relevant interest groups, or stakeholders that are involved in AVIs, using an ethical matrix [30]. This tool assists in the ethical analysis by highlighting the different perspectives and different concerns of the relevant stakeholders and incorporating them in an overall framework. Stakeholders constitute the rows of the ethical matrix (EM). The columns of the EM are represented by prima facie ethical principles that represent the different concerns involved in common morality: Wellbeing, autonomy, and fairness. Each cell constitutes the ideal situation for each stakeholder (line) as regards to that principle (column).

The main goal of the customized EM is to integrate the various ethical concerns in a single frame, allowing to identify two main kinds of ethical concerns:

- Potential conflicts (when the ideal situation for one stakeholder conflicts with the ideal situation for another stakeholder);
- Non-conformities (when the real situation, as results from the previous steps of the protocol, does not correspond to the ideal situation detailed in the EM, where all the concerns are respected).

Data resulting from the multidisciplinary analysis conducted in the previous steps can be compared with the ideal situation resulting from the customization of the EM accordingly to the specific AVI under examination. This, in turn, is useful for the final step of the protocol, where an overall evaluation of the AVI is formulated.

Step F consists of a checklist with eleven entries (Table 1). Checklists are a common tool for the ethical assessment in other fields, like scientific research with animals, allowing both self-assessment and ethical committees evaluation of animal research protocols [31,32]. The use of EMs in combination with a final checklist appears to be able to support the appraisal of the main ethical relevant aspects of AVIs under examination. This, in turn, can promote transparency and consistency of the overall assessment process.

The checklist is designed to provide an explicit outcome of the assessment process in which strengths and weaknesses of an AVI are easily spotted and can be shared and communicated.

All the steps of the protocol should be adapted from time to time to the different facility/situation, to account for the high variability in AVIs. Authors are conducting other pilot tests on different AVIs (e.g., walk-in in lemurs’ enclosures, touch pools, riding elephants, etc.) to assess the overall protocol and whether there could be common issues that can be identified for all the AVIs.

## 3. Animals, Materials, and Methods

A pilot study regarding the application of AVIP was conducted on a “giraffe feeding” interaction. This study was carried out between August and September 2017 in a northern Italian zoological park (Zoom biopark, Cumiana, Turin, Italy, 44°55’59” N, 7°25’18” E). The study was observational in nature and was made in accordance with both the ethical regulations of the participating institutions and the national regulations. The animals involved were housed at the zoo and husbandry routines were not changed or affected by the study and the ARRIVE (Animal Research: Reporting of In Vivo Experiments) guidelines were applied [33]. All visitors gave their consent for inclusion before they participated. Neither animal care license nor approval of ethical committees was therefore needed [14]. The biopark offers visitors both activities, such talks in which biologists and keepers give additional information of some target species housed in the park (e.g., penguins, Serengeti animals, and lemurs), and experiences allowing direct contact and interactions with giraffes, tortoises, and birds of prey.

**Figure 1:**
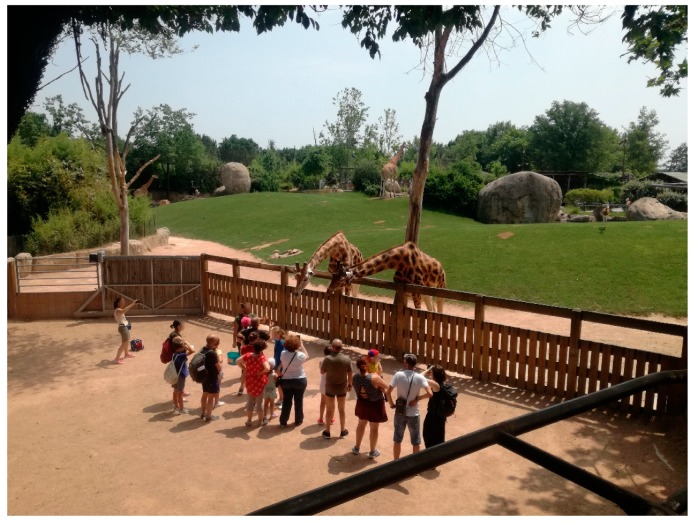
Illustrated is the area where the “giraffe feeding” took place. Visitors were allowed to get access to the outdoor area of the night enclosure.

Each “feeding the giraffes” interaction lasted 20–30 min and a maximum of 20 people could participate in each interaction after having paid an additional ticket. During the season in which the study took place, the interaction program took place twice daily (15:20 and 16:45) from Monday to Friday, and three times a day (12:15, 15:20, and 16:45) on Saturday and Sunday, provided enough people had booked it. A keeper accompanied the visitors to the outdoor area of the night enclosure, near the fence dividing the night from the day enclosure, while the giraffes were in the day enclosure (Figure 1). (For an aerial view of the giraffes’ enclosure see also [14]).

The keeper then called the giraffes near the fence so that they could lean down with their necks over the fence and with their heads within reach of a visitor standing near the fence in the night enclosure. When called, the giraffes could choose to come and interact or not, and they could walk away from the interaction at any time. As a rule, only two giraffes (Sam and Frantisek) chose to interact. The keeper told visitors to stand in a line and delivered a short talk about giraffes and giving instructions regarding how to interact with the animals during the program. Then, two visitors at a time could approach the giraffes’ heads, feed them palatable food, and pet them. The food offered during the interaction was in addition to the giraffes’ normal diet. When all visitors had the opportunity to feed, pet, and eventually take pictures of themselves with the giraffes, the interaction ended and the keeper led the visitors to exit the night enclosure.

### 3.1. Animal Welfare Assessment (Steps A to C1)

A detailed description of the animal welfare assessment of the “giraffe feeding” interaction is given in Normando et al. [14] (see also Table A1).

### 3.2. Human Outcome Assessment (Step C2 and D)

For the present pilot study, the “human outcome assessment” was tailored to include:

- A risk assessment procedure for health hazards (infectious/parasitic diseases and risk of traumas/injuries) as related to both the visitors participating in the AVI and the staff running it (step C2);
- A visitor experience evaluation (consisting of a visitor experience analysis checklist and a visitor experience survey) (step D).

Informal talks with keepers and other members of the staff were used to get their point of view in order to complete the ethical matrix in step E.

#### 3.2.1. Risk Assessment Related to Visitors and Keepers (Step C2)

The assessment was carried out following the documents proposed by DEFRA [26] and EAZA [27]. These documents are oriented to the identification of the necessary measures to eliminate or reduce exposure to hazards by means of mitigation actions. These actions are based on prevention (set of regulations or measures necessary to avoid or reduce risks—normally aimed at reducing the probability of the event occurring) and protection (all collective or individual measures aimed at eliminating or reducing a risk—they aim at reducing the extent of damage in case the event occurs).

Risk (R) is defined by the probability (P) that a hazard occurs multiplied by the magnitude of the damage (D) that can derive from the hazard if it happens.

It was therefore decided to carry out a semi-quantitative assessment where the risk (expressed by risk score—R) is defined as in the following mathematical relationship:

(1) $$R=\frac{\left(P\times D\right)}{Ki}$$

where Ki represents the prevention and protection measures.

Prevention and protection measures can be divided into three different categories:

1. **General preventive security measures**;
2. **General control measures of zoonotic risk** which are divided into five subsections (i.e., biosecurity, veterinary control, environmental hygiene, design of the exhibition areas, control measures for the risks of infection);
3. **Protective measures**.

**General preventive security measures** are those which can be applied to all types of hazard, such as training and informational activities. They can be done through verbal or poster communication, sometimes with simplified content in pictograms or signs. 

**General control measures of zoonotic risk** are measures to control the spread of zoonoses in animals and the environment. They include high standards of biosecurity and adequate veterinary control aimed at reducing or eliminating the possibility of diseases occurring in animals. 

Measures of environmental hygiene and design of the exhibition areas are those that reduce the contamination of the environment and of the people themselves. 

Control measures for the risks of infection are measures against the risks of infection related to the contamination of people and exposure to diseases (personal sanitary measures implemented by people in order to control the risk of infection, e.g., washing hands, avoid eating in the areas of animals).

**Protective measures:** Control measures related to the contamination of people or protecting them from injuries (use of PPE—Personal Protective Equipment).

Analysis of the existing preventive and protective measures and other actions that could be implemented was conducted using an ad hoc checklist, asking the presence or absence of each measure (Table A2).

In the checklist, YES was recorded when the researcher saw/heard the staff member performing the action (e.g., informing the public on what not to do during the interaction) or if there was any visual evidence about structural requirements (e.g., there were adequate signs displayed advising visitors not to smoke, or there was an adequate number of soap dispensers) or if there any documentary evidence (e.g., if the veterinarians perform zoonotic risk analyses, or if the keepers have continuous training on different matters) NO was chosen when these requirements were not met suggesting any action was taken, and in absence of visual and documentary evidence, following visual inspection and verification of the existent documentation.

Using the checklist results, the risk assessment procedure was carried out on five different phases:

**Phase 1: Hazard identification.** In this phase, an exhaustive list of potential biological and physical hazards was created. The list of the zoonotic agents to be included was based on scientific reports of diseases in species involved in AVIs [34,35,36,37,38,39,40,41,42,43,44,45,46,47,48,49,50,51,52,53].

**Phase 2: Hazard characterization (P).** It concerns the likelihood of people being exposed to a specific hazard. The categories are rare, unlikely to occur, probable, and very likely to occur/certain event and they take numerical values on an ordinal scale from 1 to 4, taking into account the existing preventive measures. Rare describes an event very unlikely to happen with <5% chance of it happening, unlikely is an event not expected to happen but it may present with a probability ranging from 10% to 30%, probable is expected to happen with a probability ranging from 30% to 95%, while a very likely/certain event will occur with a probability >95%.

**Phase 3: Exposure assessment (D).** It concerns the magnitude of the damage. The categories lower, mild, serious injury and death take numerical values on an ordinal scale from 1 to 4, taking into account the existing protective measures. Lower is referred to a minor damage resolvable with on-site medical treatment (first-aid kit), mild is a moderate damage that requires at least a few days of prognosis, serious is damage requiring more than 30 days of prognosis or that could be permanent while death is relating with a poor prognosis. 

**Phase 4: Risk characterization.** On the basis of the outcomes of phase 2 and phase 3, the risk was calculated (Table A3). The resulting risk categories (Table A4) were then defined by verifying the opportunity to implement certain mitigation activities and their urgency.

**Phase 5:** This is a reiteration of phases 2–4 after an analysis of the additional preventive and protective actions in the case they must be implemented (Ki) to mitigate the risk. 

#### 3.2.2. Visitor Experience Assessment (Step D)

A preliminary analysis of the educational aspects connected with the “giraffe feeding” interaction was needed in order to implement step D of the protocol. While doing the preliminary behavioral observations, the researcher also collected the information needed for the design of the questionnaire. During the preliminary behavioral observations, notes on what was said by the keepers running the AVI were collected, and from those notes a standardized checklist, called preview visitor experience analysis checklist (Table A5), was created. The information gathered using this checklist was necessary for the design of the ad hoc questionnaire to be used in step D and to perform further steps of the protocol. Besides allowing knowing what kind of factual information was provided to visitors during the interaction, the preview checklist also investigated if adequate behaviors to promote animal welfare and wildlife conservation were suggested by the staff. This is important because scientific literature agrees that information alone is not sufficient in motivating people to adopt new behaviors [54,55].

An ad hoc questionnaire was designed, including items specifically created to assess interaction outcomes and to provide data for the other steps of the protocol. Methods and models taken from other fields (as word association and Kano model, see detail below) were included in the survey questionnaire. This was done to assess their results when applied in a different context and to compensate for the scarcity of terms of comparison in the scientific literature concerning the educational and emotional outcomes of AVIs involving direct contact in zoos [56] and in aquariums [57,58].

Three versions of the questionnaire were designed: A pre-interaction version (preQ) and a post-interaction version (postQ) to be administered to visitors before and after the AVI, and a third variant to be administered to general visitors (genQ) (see for their English version in the supplementary materials, Supplementary 1–3, Table S1).

The preQ version of the questionnaire included three open-ended questions, the postQ included fifteen items, and the genQ thirteen items. Details on the questionnaires are given in Table A6 and the English translation of the questionnaires is included in the supplementary materials. 

In all the versions of the questionnaire, a word association task investigating respondents’ perceptions about giraffes was included at the beginning of the questionnaire to be sure that the following items did not influence the association. Respondents were asked to write down the first three words [59] that came to their mind when they thought of a giraffe. Three blank spaces to fill in were provided to the respondents. The frequency of elicitation was related to the importance of a concept in consumers’ mind (i.e., visitors in the case of zoos) [60].

In order to investigate respondents’ experience, they were asked their reasons for joining or not joining the AVI, and if they already joined it, what were their expectations about it. Respondents’ satisfaction was investigated asking likelihood of recommendation, the perceived value of the experience, and satisfaction on pre-information experience. The pre-information experience consisted of the details given to visitors by the staff before the interaction and whether it improved visitors’ autonomy in the choice of joining the interaction or not and in their awareness during the experience.

Likelihood of recommendation was investigated accordingly to the Net Promoter Score [61]. Respondents scored on a 10-point scale [62] how likely they would recommend to a friend to join the “giraffe feeding”. 

A Net Promoter Score value was then calculated using the following formula:

Net Promoters Score = % promoters − % detractors(2)

According to Reichheld [61], promoters (score 9–10) are loyal enthusiasts who will keep buying the service and recommend it to others. Passives (score 7–8) are satisfied, but unenthusiastic customers. Detractors (score 0–6) are unhappy customers who are likely to comment negatively on the service to others. In this study, the service was the AVI with the giraffes and the customers were the visitors. 

A deeper satisfaction analysis was done using a Kano model [63] and targeting four different attributes that could be present in interactions:

- Direct contact with the animal;
- Information about the specimens involved in the interaction (age, gender, biographical info, etc.);
- Information about convenient behaviors for the conservation of the species involved;
- Instructions given on how visitors should behave in order to not compromise the welfare of the involved animals.

The Kano model was developed in the commercial sector in order to categorize the attributes of a product or a service depending on how well they satisfy customer needs and preferences. An attribute is something that characterizes the product/service that can be present or absent, or differ. For example, if we consider a mobile phone, attributes to be analyzed can be the price, the color, the dimension, the size of the internal storage, the quality of the camera, etc.

Although used for quality management also outside the commercial sector [64,65,66], up to now the model has been used in zoos only for product or service development and customer satisfaction [67].

**Figure A1:**
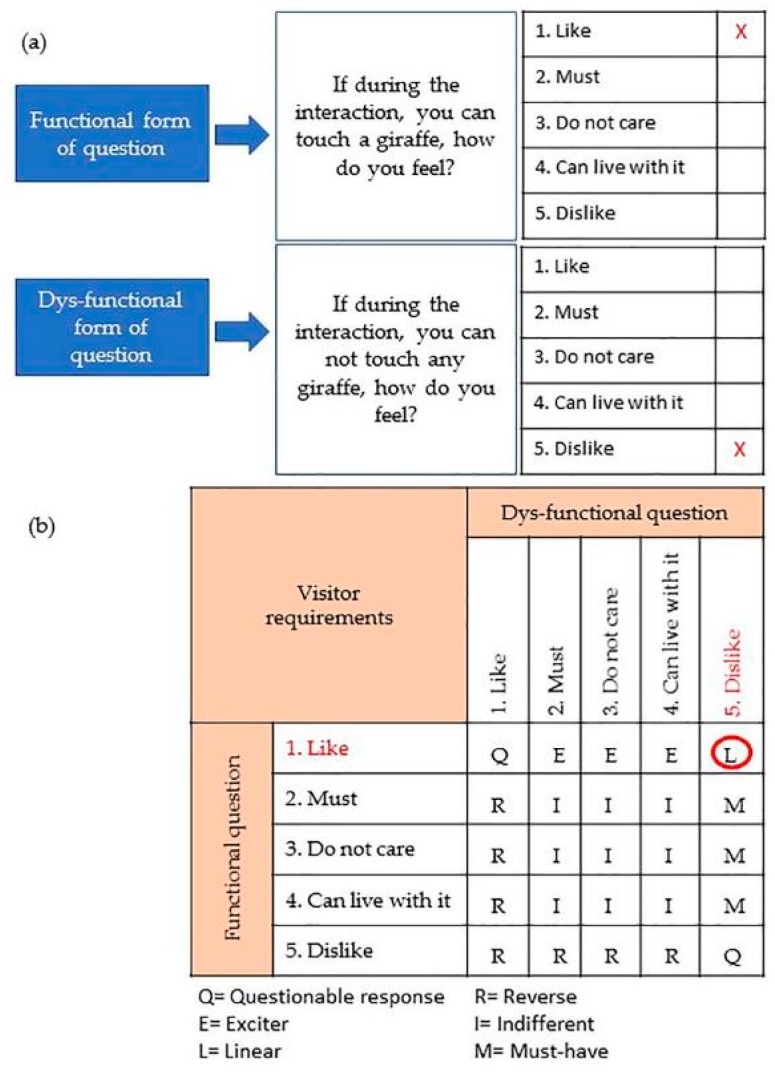
Kano Analysis (**a**) questionnaire format and (**b**) evaluation table for the classification of the results. Two red crosses indicated the hypothetical answers of a respondent in the questionnaire and a red circle mark the successive classification.

In order to see if this model can be adapted to investigate the attributes of an AVI with an educational and ethical perspective, a Kano model like grid was included in the postQ. For each attribute under investigation, two versions of the same question (one functional and one dysfunctional) were asked and four answer options were provided for each (Figure A1a). The attribute is then classified for the customer using the combination of the answers given to the functional and the dysfunctional version of the question using a dedicated table (Figure A1b).

An attribute can thus be categorized as:

- E (Exciter): Indicating that this attribute is an attractive respondent’s requirement;
- I (Indifferent): Meaning that the respondent is neutral in regards to this attribute;
- Q (Questionable response) stands for an ambiguous result, probably due to mistakes;
- R (Reverse) means this attribute is not wanted, and the respondent expected the opposite;
- M (Must-have), indicating a critical quality requirement from the respondent’s viewpoint;
- L (Linear), meaning that the respondent’s satisfaction is directly proportional to the level of fulfillment.

The average impact of each attribute on the satisfaction or dissatisfaction of respondents can then be calculated using the Customer Satisfaction coefficient (CS coefficient). This coefficient is indicative of how strongly an attribute may influence satisfaction or dissatisfaction [68]. CS coefficient ranges from −1 to +1, when it is close to the external values it means that the feature has a high influence on customer opinion and when it is adjacent to 0 it means the attribute has a low influence. For each attribute, the extent of satisfaction and dissatisfaction must be calculated with different formulas.

To calculate the extent of satisfaction this formula can be used:

(3) $$+\;\frac{E+L}{E+L+M+I}$$

The nearer the value is to +1, the higher the satisfaction will be if the attribute is present during the interaction and vice versa if close to 0. To calculate the extent of dissatisfaction, the formula is the following:

(4) $$-\;\frac{L+M}{E+L+M+I}$$

If the coefficient approaches −1, the influence on customer dissatisfaction is especially strong. A value of about 0 signifies that this feature does not cause dissatisfaction if it is not met [69].

##### (1) Data Gathering

One of the authors distributed the self-administrated questionnaires to visitors. Data collection began on 25 August 2017 and continued until 24 September 2017. School groups, staff, and zoo volunteers were excluded from the study. 

The preQ was administered near the interaction’s exhibit to visitors who were going to participate in the “giraffe feeding” when they arrived at the location 15–20 min before the experience. An identification number was given to every respondent of the preQ to match them with the postQ. The postQ was distributed at the end of the interaction only to visitors who agreed to participate in the first phase of the survey.

The genQ was administered for 14 days from 25 August to 15 September for one hour per day around 16:00. Potential survey respondents—zoo visitors aged 18 years or above—were selected using systematic sampling (every 3 units: Single visitor or group). Visitors were gently stopped near the exit of the zoo and the scope of the questionnaire was explained. Thus, the sample included both visitors who joined an interaction on the day of the survey and those who did not. 

The questionnaires were anonymous and participation voluntary.

##### (2) Data Analysis

Data were screened for missing variables. Among genQ respondents, those who did not participated in any AVI (henceforth called “NPV”, Non-Participating Visitors) were identified and used for further analyses as detailed below. Chi-squared analyses were used to investigate whether groups (preQ/postQ vs. genQ) differed significantly on any demographic and independent variables. Moreover, a chi-squared contingency test was done to investigate if respondents who joined an interaction—giraffes, tortoises, and falcons’ interactions—(preQ/postQ plus genQ who participated in AVI) had distinctive characteristics (sex, educational level, pet ownership, natural childhood, first visit, age) than NPV.

Data about reasons to participate or not to participate were grouped by theme in categories and descriptive statistics were performed on them. The same analysis was done on the reason why respondents thought that the AVI adds value to their day at the zoo. 

The answers of each respondent to the Kano model were classified by the combination of the answers to the functional and the dysfunctional questions and then classified using a dedicated table (Figure A1b). For each attribute, the combination chosen by the majority of the respondents defined the classification of the attribute. The frequency of respondents who said Linear, Exciter, Must-have and Indifferent were used to calculate the CS coefficient with the Formula (3) for satisfaction and (4) for dissatisfaction. 

The likelihood of recommendation was used to calculate the Net Promoter Score with the Formula (2) as indicated in Section 3.2.2.

A lemmatization was performed on the world association task results, grouping together the inflected forms of a word so that different words were able to be analyzed as a single term (e.g., high, height, and the highest were grouped together). After this process, words elicited were divided into 2 groups according to the “emotional” load they entailed (sympathy, sweetness, beautiful, funny, etc.) or not (mark, neck, skin, high, Africa, etc.). Three different researchers did the grouping and then the three classifications were compared and a complete agreement was found. Then, descriptive statistics were performed and a McNemar test was used to compare the words elicited by the same respondent before and after the interaction (preQ vs. postQ). In this analysis the words were compared in the order they were written by the respondents: Thus, the first word given in the preQ with the first word in the postQ, and so on. When a different number of words were given before and after the data were eliminated, a chi-squared test was performed to investigate if the number of elicited “emotional” and ‘non-emotional’ words differed between genQ respondents who joined interactions and NPV and between preQ answers and NPV ones. Chi-squared tests were also used to compare the availability to give the email addresses between the respondent of the postQ and NPV.

### 3.3. Final Overall Ethical Assessment

#### 3.3.1. Ethical Analysis (Step E)

A customized ethical matrix (EM) was developed for Step E following a top–down approach, where an ethical expert fills in the cells [30,70]. In this case, cells were filled in by researchers, considering data gathered from the other steps of the protocol. The identified interest groups having an ‘ethical standing’ were the giraffes, visitors, and the zoo staff. As shown in Table 2, since not all four of the zoo’s giraffes participated in the activity with visitors, those not participating have to be considered as a separate group because they can have different concerns. As a result, we have an interest group composed of the giraffes involved in the activity (ZG), who are directly affected by the interaction in terms of welfare and quality of life, and a second group including those who are not involved (G). In addition, we included a third interest group, the wild counterpart of the zoo giraffes (W), because part of the money gained from AVIs is invested in conservation programs. Visitors are made up of people who decide to participate in the interactive activity (A) and, separately, people who do not (V). Finally, since the different zoo staff members had different roles and concerns over the AVI, we considered separately keepers (F), the heads of education, research and conservation departments, and the manager or director (G), the veterinary staff (H), and finally, the zoo (I).

Each cell of the ethical matrix was then populated considering the ideal situation about the AVI in which the relevant concerns were respected for the relevant stakeholders. The ideal situation of each stakeholder was then weighed against that of any other to identify possible internal conflicts. Moreover, the ideal situation for each stakeholder was compared with the actual situation of the AVI emerging from data collected from the previous step of the protocol that were used to represent the actual situation. In particular, data obtained from the animal assessment (step A to C1) of the AVIP were compared with the cells in which the giraffe’s interest groups were investigated, whereas data obtained using the human outcome assessment (step C2 and D) were compared with the other relative interest groups in the ethical matrix.

#### 3.3.2. Final Assessment (Step F)

A standard Step F, as described in Section 2.3, was applied in the “giraffe feeding” study.

## 4. Results

### 4.1. Animal Welfare Assessment (Step A to C1)

No concern resulted from behavioral analysis and a low risk was identified by risk assessment (for more details see Normando et al. [14]).

### 4.2. Human Outcome Assessment

#### 4.2.1. Risk Assessment Related to Visitors and Keepers (C2)

Analysis of the existing preventive and protective measures—the results of the checklist for the analysis of the risk mitigation measures were suitable to guarantee adequate safety standard of people during interactions. The only items for which a negative answer was recorded were:

- The possibility to store their personal items in the safe area (items 1.2, 5.5);
- Washing and disinfection of the visitors’ hands after the interaction (items 5.11, 6.4);
- Reminding the visitors with adequate signs of the rules during the interaction (item 1.12).
- The existence of a service access point differentiating from visitors entrance or exit (item 5.4).
- The use of PPE (items 7.1, 7.2).

**Phase 1: Hazards identification:** (1) Anthropozoonoses: The main zoonotic agents (both bacteria and fungi) were identified on the basis of literature findings on main diseases in giraffe and in other animals reported in zoo human–animal interactions (Table A7 reports a detailed list of the zoonotic agents and the relative references). Other zoonotic diseases where a giraffe has a little relevant epidemiological role (e.g., host with little probability to transmit the disease) and all those diseases transmitted by arthropod vectors were not considered, because the interaction with animals does not increase the risk of contracting the disease. (2) Injuries: The visitor and staff involved in the interaction can suffer traumas due to neck blows. Contact urticaria syndrome due to an immediate-type hypersensitivity to animal proteins can be a problem in people exposed to animals [71,72].

**Phase 2: Hazards characterization:** The results of the hazards characterization are detailed in Table 3 and expressed in terms of probability (P).

**Phase 3: Exposure assessment:** The results of the exposure assessment are detailed in Table 3 and expressed in terms of damage (D).

**Phase 4: Risk characterization:** The results of the risk characterization are detailed in Table 3 and expresses in terms of risk score (R) and risk rating (RR) obtained with the existing control measures.

**Phase 5: Risk characterization:** Predicted with the additional control measure that could be implemented are detailed in Table 3.

#### 4.2.2. Visitors Experience Assessment (D)

A total sample of 173 visitors answered the questionnaires. A summary of the demographic information and other independent variables collected in the preQ and postQ from respondents (*N* = 67) and from visitors interviewed nearby the exit of the zoo, genQ (*N* = 106), is presented in Table A8.

Only three variables differed between pre/postQ and genQ respondents: Visitors with friends (*p* = 0.016), with an annual ticket (*p* = 0.01), or who had already visited the zoo in the past (*p* < 0.001) were less likely to take part in the “giraffe feeding” AVI. 

Number of past visits to the zoo differed also between respondents who had joined any AVI and those who had not, the former being more likely to be at their first visit (χ^2^ = 14.100, df = 1, *p* < 0.001)

A total of 135 different motivations to participate in the “giraffe feeding” were mentioned by respondents in the preQ. All the motivations were grouped into seven categories, the most frequently mentioned being the research of contact and proximity with the giraffe (*N* = 29; 21.5%). Among respondents of the genQ, only 15% (*N* = 16) of them joined the “giraffe feeding” on the same day of the survey and the great majority (76%, *N* = 81) of the interviewed did not participate at all in any AVI on that day. A total of 79 reasons were mentioned by respondents for not having joined an AVI. These answers were grouped into eight categories and the most frequently mentioned one was that “it was not possible to schedule” (*N* = 20; 25.3%). Table A9 and Table A10 in Appendix A give further details on the stated motivation for joining or not joining the AVIs.

After the AVI, respondents were asked if they would recommend the experience to a friend on a scale of 1 (“absolutely not”) to 10 (“absolutely yes”) value. The great majority of the sample (73.1%, *N* = 49) answered “absolutely yes” and nobody answered “absolutely no” (mean 9.31; median 10). Therefore, according to the Net Promoter Score [61], 82% (*N* = 55) could be considered as promoters, 11% (*N* = 7) as passive and only five respondents (8%) as detractors, and the Net Promoter Score was 74%. 

A considerable 97% of respondents (*N* = 65) declared that the AVI added value to their visit to the zoo and 55 respondents (82.1%) gave a reason for their statement. The answers were grouped into four categories (Table 4) and the most mentioned category was the one labeled “experience/emotion”.

The pre-information experience was said to be satisfactory by 91% of the sample (*N* = 61). The most frequently mentioned issues were that the information was comprehensive, and the guides were well trained. 

The Kano model result analysis (Table 5) showed that all the attributes investigated were a “Linear” requirement for most of the respondents. However, “direct contact with animals” was exciter for 42% of the respondents, meaning, it was an attractive feature providing them with high satisfaction because it was unexpected.

**Figure 2:**
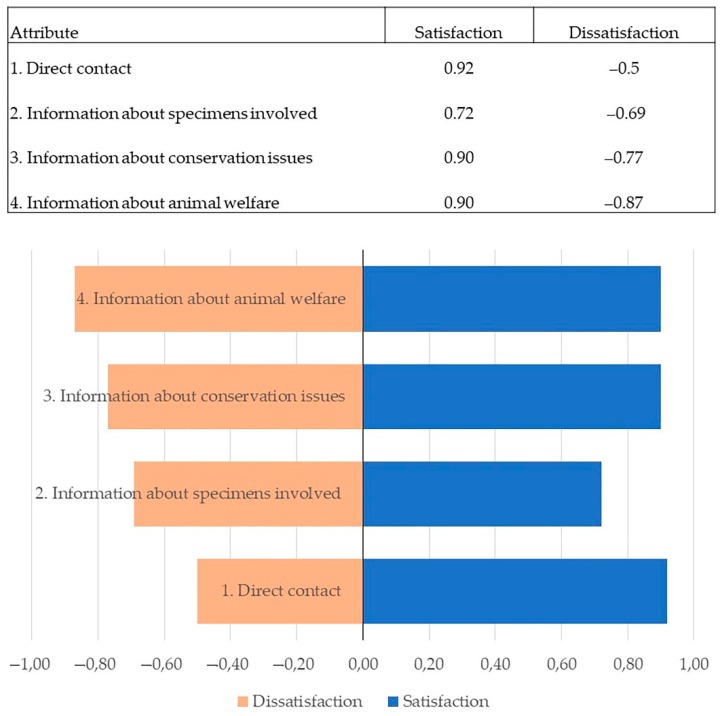
Customer Satisfaction coefficient (CS) calculated for the four attributes of the interaction activity “giraffe feeding”. Evaluating the causes for satisfaction (right part of the graphic, blue stripes): The closer the value is to +1, the more the presence of that attribute influences respondents’ satisfaction. On the left side of the figure (orange stripes): The closer the value is to −1, the more that attribute influences respondents’ dissatisfaction if not present in the interaction.

The calculated CS coefficients for respondents’ satisfaction or dissatisfaction are shown in Figure 2.

To investigate if respondents were more proactive toward conservation projects after joining an AVI, the disposition to give their email address to be involved in a future conservation project of the park was investigated. A chi-square test was performed to compare visitors’ answers in postQ and in genQ (excluding the genQ respondents who had joined an AVI on the day of the survey), but only a weak tendency to differ was found (X² = 3.640; *p* = 0.056), with visitors interviewed after the AVI being more willing to give their email address than NPV.

**Figure 3:**
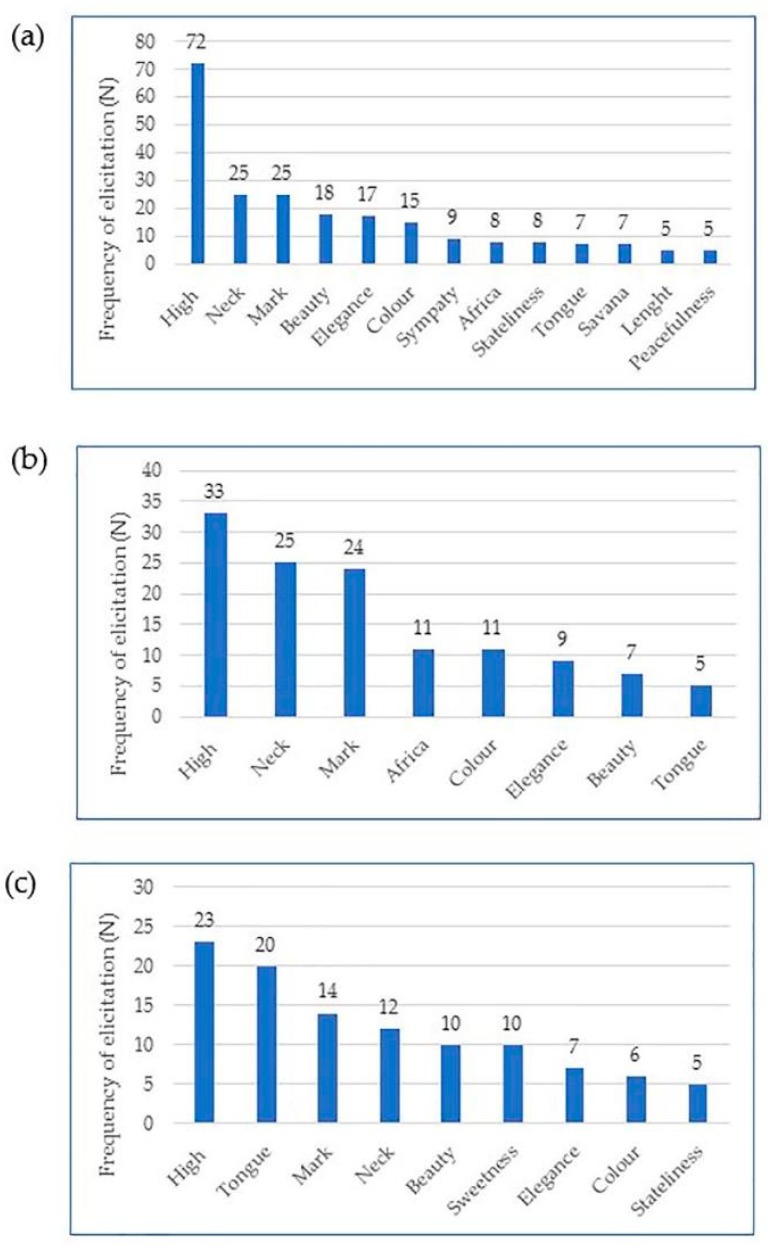
Frequency of elicitation of words in general visitors (genQ) (**a**), pre-interaction version (preQ) (**b**), and post-interaction version (postQ) (**c**). In the three graphics, all the words (after lemmatization) that were mentioned by five respondents or more are included.

The total number of valid elicited words in the ‘giraffe’ word association test was 291 for the genQ, 193 for the preQ, and 167 for the postQ. An average of 2.71 words associations were given by respondents, with all the respondents interviewed giving at least two words. Words given by more than five respondents, as divided by version of the questionnaire, are shown in Figure 3.

A significant difference was found in the amount of ‘emotional’ words elicited among the same respondents before and after the AVI (McNemar’s test; *p* = 0.009; *N* = 165), but in 11 cases an emotional word in the preQ was changed into a non-emotional word in the postQ. In the following analysis, an almost significant difference was found when comparing the respondents of the genQ who joined an AVI and who did not (X^2^ = 0.365; *p* = 0.546), but no difference was found comparing the words elicited by the respondents in the preQ and in the genQ (considering who did not join an AVI) (X^2^ = 1.711; *p* = 1.191).

### 4.3. Final Overall Ethical Assessment

#### 4.3.1. Ethical Analysis (E)

The ethical analysis enabled to compose all the different data gathered from the other steps of the assessment process (steps A to D) into an overall framework, allowing an ethical assessment of the value of the AVI, which is then finally elaborated in the eleven entries checklist in step F. Two distinct phases composed the ethical analysis of step E. First, the customized EM, enabling to represent the ideal situation in which needs and values of all the relevant stakeholders were respected, was populated (Table A11). Second, the results of the previous steps of the protocol were compared with each of the corresponding cells of the EM. Ethical concerns, both in terms of potential conflicts between different cells and of concerns emerging from non-conformities, were then identified. Regarding potential conflicts, in the “giraffe feeding” AVI, no animal welfare risk was detected and no conflicts elicited by the desire of visitors to be near or even in contact with animals [73] were identified. Other sources of conflicts in the EM customized for the “giraffe feeding” AVI were not detected. Concerning non-conformities, results of the ethical analysis, focusing on the content of the most relevant cells for each stakeholder, highlighted the following.

**Zoo giraffes participating in the AVI.** As the animal risk assessment reported overall low risk for welfare and the behavioral observations did not detect any change in behavior that could be revealed a stressful situation for the animals, the wellbeing of the giraffes participating in the AVI was respected (ZGW). Moreover, indications about how to act during the AVIs were correctly given by the staff and were found satisfactory by the great majority of the respondents (91%). This information is important to instill in the visitors the awareness that they are coming into contact with a sentient being and to teach how to respect animals. 

The results of the enclosure analysis highlighted that the enclosure was suitable to host the interactions and it allowed giraffes to choose to interact or not with the visitors. This situation permitted a good degree of control on the environment and an adequate freedom of behaviors (ZGA). 

The fact that all the giraffes remained together in the usual enclosure, so that the usual opportunities for positive welfare outcomes were available to all the animals, was interpreted as corresponding to the fairness ideal situation for participating giraffes (Table A3). As a whole, no ethical concern was identified for this stakeholder.

**Zoo giraffes not participating in the AVI.** Two of the four giraffes never participated in the AVIs. No differences in behaviors were detected in these individuals between the interaction and the control episodes suggesting that wellbeing was respected (GW). Therefore, it is likely that they really did not wish to take part in the interaction. Their behavioral freedom was also likely to be safeguarded (GA). The same said for participating giraffes can be said for fairness here. No ethical concern was identified here, too. However, if further evidence suggests that the non-participating giraffes are avoiding participating because they are inhibited by the presence of a dominant individual, it would be considered an ethical concern due to infringement of the ideal situation in cell (GF). 

**Wild giraffes and environment.** Part of the money from the AVI extra tickets was devolved to conservation and the AVI seemed to have a positive effect on conservation attitudes as seen by a weak tendency to increase in e-mail addresses given after the AVI. Moreover, as highlighted when describing results about visitors participating in the AVI, an indirect effect for the environment could be detected because respondents after the AVI were more likely to link “emotional words” to the word giraffe than before [74]. 

**Visitors participating in the AVI.** Since the results of the risk assessment related to visitors highlighted low and medium risks of contamination, an ethical concern was identified related to their wellbeing (AW). Their physiological wellbeing was at risk to be impaired by factors because visitors could not store their personal items in a safe area and no washing stations were provided to wash their hands after the interactions. Safety indications were given by the staff, but no written signs reminded these indications to visitors during the AVIs. Their psychological wellbeing, on the other hand, was respected: The AVI allowed proximity and tactile contact which was the first reason that respondents mentioned to join the “giraffe feeding” and the CS coefficient confirmed that direct contact with animals had a high influence on respondents’ satisfaction. (AW). Furthermore, 91% of the visitors participating in the AVI were pleased by the information given by the staff before the AVI about what to expect. Autonomy for visitors appeared to be respected (AA). The interaction activities were not included in the zoo’s ticket and were an extra cost to the visitor. Irrespective of the additional price requested, this was not perceived to be expensive with only 14% of respondents replying not to have joined the AVI because of the price. Net Promoter Score was 74%, and 97% of respondents stated that the interaction added value to their day at the zoo. As far as fairness was concerned (AF), even if minimal information about the biology of the animals or the conservation of the species was given during the interaction, an emotional outcome was detected by comparing the words elicited in the word association task: Visitors after the AVI were more likely to link ‘emotional words’ to the word giraffe than before.

**Visitors not participating in the AVI.** Visitors could choose not to participate in the AVI if they were not interested (19% of the interviewed) or were not willing to pay an extra ticket (14%), so they were free to choose (VA). As far as fairness was concerned, visitors not participating in the AVI had alternative options to receive information about the animals. The zoo offers visitors a wide range of free talks in which such information is provided. This notwithstanding, about 35% of the respondents said they had no opportunity to join the AVIs, causing some concern about fairness as applied to them (VF).

**Keepers (involved in AVIs).** Keepers appeared to be in general well satisfied of their participation in AVIs and confident that the animals they cared for received a good standard of welfare during the AVIs, so their psychological wellbeing appears to be respected (KW). Regarding their physiological wellbeing, albeit keepers were correctly instructed on the use of disinfectants and on the implementation of biosecurity activities necessary due to the presence of visitors, the risk assessment showed that the contamination risks during the interaction were low and medium, so a concern was found. It is important to consider that during the AVIs keepers are responsible for the safety of visitors that participate in the AVI and the quality of their experience and for the welfare of the animals at the same time. This responsibility could be taxing, so it is crucial to monitor this aspect when assessing AVIs. In the case of the “giraffe feeding” AVI, keepers did not voice any concern about this issue when talking to the researchers (KW and KF). 

**Management staff.** The staff responsible for managing and coordinating the AVIs have many responsibilities around all the three areas of assessment (MA): Both the animal welfare and the quality of the experience for visitors has to be guaranteed by coordinating efforts, and the ethical assessment is based on the outcomes of these efforts. It is therefore crucial that the management staff should have the required resources and well trained staff to ensure that AVIs are conducted correctly (MW and MF). In this AVI no concern was identified with regard to management staff. 

**Veterinary staff.** As in the case of keepers, the risk assessment related to the veterinary staff has shown that the contamination risks are low and medium. The professional activity of the veterinarians who regularly monitor the animals involved in the interaction is of paramount importance to safeguard both animal welfare and visitor security; therefore, both his/her wellbeing and his/her access to fair working conditions (VSW and VSF) have to be monitored. The role of the veterinarian, as in the case of the other people who can make decisions affecting the AVIs, is crucial regarding all the three areas of assessment that a discretionary power should be given to them (VSA) regarding how to monitor and safeguard animal welfare (not only during AVIs). Furthermore, they should be allowed to contribute to the quality of education and in the implementation of biosecurity activities. 

**Zoo.** AVIs were often sold-out, this means that they contribute to the economical sustainability of the facility (ZW and ZF). Furthermore, as shown by the results of the questionnaires, respondents were satisfied and well-disposed to recommend the experience to other people, thus contributing to improve the good reputation of the facility (ZW and ZA). 

#### 4.3.2. Final Assessment (Step F): Alert and Good Practices

In the final overall assessment, the target AVI had a ‘yes’ in entries 1, 3, 4, 7, 9, 10, 11. No answer could be given to entry 2, because step B was not performed (for further details about the reasons, see [14]).

## 5. Discussion

The present paper presents a six-step protocol aimed at identifying possible ethical concerns of a target animal–visitor interaction (AVI). The protocol was developed on the basis of Padua University’s Ethical Review Process [75] and includes three areas of assessment: Animal welfare, human outcome, and overall ethical assessment. The whole protocol was tested to evaluate an AVI program involving giraffes in a northern Italy zoo.

The innovative aspect of the AVIP, following WAZA recommendations, appears to be that, unlike other protocols aiming to identify concerns on a single aspect, it does not rely on a single tool, but consists of a six-step process encompassing detailed areas of assessment. The six steps of the protocol thus allow evaluating, within a comprehensive ethical framework, the main aspects of an AVI: Animal health and psychological welfare, health hazards and other risks for people, emotional impact, and educational and conservation mindedness outcomes. 

In the “giraffe feeding” AVI, the welfare of the giraffes was not found to be negatively affected by the AVI in steps A to C1, so the ideal situation of their wellbeing as regarding the interaction corresponded with the actual situation and did not necessitate discontinuing the AVI. A different scenario would arise if many more people were interested in joining the AVI. Respect for fairness as applied to visitors and concerning the right of all visitors to participate in an AVI (VF), if they wanted to, would then be likely to conflict with the participant giraffes’ well being (ZGW). A higher density of petting zoo visitors has shown to negatively affect the involved animals [12]. 

When the results of the human outcome assessment were compared with the EM ideal situation, a more complex pattern emerged. An overall high level of satisfaction was detected among visitors participating in the AVI using different methods, even if some barriers to join the interactions were also identified. Studying visitor satisfaction is important to understand visitors’ points of view and to improve conservation mindedness [76,77]. AVIs appear to fulfill a well-known human need of contact with animals [78,79,80] and to produce an emotional outcome, as assessed using a words association task. While food and consumers’ preference research has used word associations [81,82,83], to our knowledge, no previous research has investigated the use of word association to study visitor’s perception in zoological facilities. In general, the direct contact with the animals during the interaction is a critical issue in all AVIs. On one hand, it greatly increases visitors’ satisfaction [56], whilst on the other hand, it is linked to the risk of contamination [50,84] and can be a threat to animal welfare [85]. In general, allowing “direct physical contact between humans and animals in a demonstration for the sole purpose of entertainment, where there is no accompanying demonstrable educational value” is deemed questionable at least [86]. Therefore, other crucial aspects worth investigating, apart from animal welfare and risk for both, animals and humans, are the education and conservation mindedness outcomes of the AVI. 

During the “giraffe feeding”, specific information about conservation was not provided, but the study found out that the AVI had a positive emotional effect on respondents. Therefore, it is important that further studies are conducted to assess the effects of explicit educational and conservation contents on the emotional impact on visitors, and thus on their conservation mindedness. As Moss and Esson [87] point out, good intentions in the education area are not a guarantee of a good outcome, but can also be detrimental. 

Furthermore, the situation concerning visitors’ autonomy was twofold. On one hand, the great majority of respondents declared they were satisfied with the information given by the staff before the interaction. On the other hand, no information was given to visitors about animal welfare and on how to behave in order to avoid distress to the animals during the AVI. In the case of the target AVI, where animal welfare did not result to be negatively affected by the AVI, this lack of information is likely not to have caused any harm to visitors’ autonomy. In general, however, it is important to give information to visitors about how to behave, allowing them the possibility to choose to avoid behaving a certain way in order not to stress the animal.

Another concern regarded the risk assessment related to visitors’ health. In the AVI under examination, a low to medium risk for visitors’ health was detected because no washing stations were available to wash their hands after the interaction, and no dedicated room to store personal belongings before the activity. In general, because the human safety risk evaluation depends on the possibility of the emergence of new hazards connected to epidemiology and is linked to the ways in which the interaction intervenes, the analysis should be repeated whenever these hazards are revealed, thus making permanent veterinary surveillance and the involvement of veterinarians and keepers in the management decisions concerning interaction, essential. However, it is worth noting that risk assessment concerning contamination hazards originates from a different context (the food industry, see, e.g., [88]), where some of the structural features (changing rooms, place to store personal belonging before the activity, hand washing stations after it) are more often common practice. In the case of zoos, aquaria, and other facilities offering AVIs, these structural features are seldom present. When restructuring or planning a facility from scratch, it can be useful to be aware of the possibility to add such features.

The final assessment checklist enables to summarize strengths and weaknesses of the AVI. The concerns that have been already discussed can be explicated in terms of what the features are (animal welfare, safety, emotional, educational, conservation outcomes, ethical assessment) in which the AVI is lacking and thus needs addressing, allowing, as the final outcome of the application of AVIP, a comprehensive framework of the quality of the interaction and suggestions for improvements.

## 6. Conclusions

The AVIP presented in this study is designed to evaluate AVIs on an integrated level, using a transparent analysis of the main aspects involved, including all the stakeholders and the potential conflicts of values. Unlike other protocols aiming to identify concerns on a single case analysis base, the AVIP does not rely on a single tool, but consists of a six-step process encompassing dedicated data acquisition and a specific ethical assessment. Behavioral and physiological analyses are fed into risk assessment procedures and used to assess animal welfare and health risks; questionnaires explore people’s experience and educational and conservation oriented outcomes; and an ethical evaluation is then conducted using an ethical matrix to identify ethical concerns, highlighting the weak points of the AVI to be summarized in the final checklist. In the context of the ethical assessment, the ideal situation represented by the populated version of the customized matrix is compared with the actual AVI, represented by results of the behavioral and physiological analysis, of the risk assessment procedures and of the surveys. Both non-conformities and cases in which the ideal situations of two different stakeholders conflict between them are considered ethical concerns and need to be addressed. In the case of the “giraffe feeding” AVI used to test the AVIP, no concern was identified for the welfare of the giraffes, whereas a suggestion for improvement (in case of restructuring) was identified for further reducing health hazard for people (i.e., provision of a place to store personal belonging before the AVI, and of hand washing stations after it) and for monitoring conservation information content. 

The protocol shows promise to be useful for zoos and other facilities, allowing to assess the overall value of their AVI programs, and to communicate their commitment to both animal welfare and education/conservation goals to the public.

## Figures and Tables

**Table 1:** Step F checklist.

N.	Entry	YES	NO
1	A behavior analysis performed correctly ^1^, did not identify any behavioral sign suggesting welfare issues (Step A).	☐	☐
2	An analysis of physiological parameters (endocrine or others), conducted correctly ^1^, did not identify any behavior sign suggestive of welfare problems (Step B).	☐	☐
3	Only a negligible or low risk of welfare health was detected in the risk assessment analysis of physiological parameters (Step C1).	☐	☐
4	No critical issues were detected when conducting an accurate ‘management and enclosure analysis’ within the welfare risk assessment (Step C1).	☐	☐
5	A negligible or low risk was detected for the health/welfare of the people (visitors and staff) in the risk assessment analysis (Step C2).	☐	☐
6	During the AVI, indications are given to increase awareness about wildlife conservation and animal welfare, and to promote sustainable behaviors among visitors (Step D).	☐	☐
7	The visitor experience analysis detected a positive emotional impact (Step D).	☐	☐
8	The visitor experience analysis detected a positive educational impact (Step D).	☐	☐
9	The visitor experience survey detected a positive impact on the conservation mindedness and/or animal welfare awareness of the visitor (Step D).	☐	☐
10	An ethical evaluation was done to highlight possible conflicts (Step E).	☐	☐
11	If any ethical concern was identified with the AVI, the zoo staff is working toward a solution (Step E).	☐	☐

^1^ Following recognized scientific standards.

**Table 2:** Customized ethical matrix.

Respect for	1. WELLBEING	2. AUTONOMY	3. FAIRNESS
ZOO GIRAFFES PARTICIPATING IN THE AVI	Physiological and psychological welfare (ZGW)	Behavioral freedom (ZGA)	Intrinsic value (ZGF)
ZOO GIRAFFES NOT PARTICIPATING IN THE AVI	Physiological and psychological Welfare (GW)	Behavioral freedom (GA)	Intrinsic value (GF)
WILD GIRAFFES AND ENVIRONMENT	Species and biodiversity conservation (WW)	Freedom from human intervention (WA)	Respect for the worth of every individual (WF)
VISITORS PARTICIPATING IN THE AVI	Physiological and psychological welfare (AW)	Self-determination (AA)	Fair treatment (AF)
VISITORS NOT PARTICIPATING IN AVI	Safety and psychological welfare (VW)	Self-determination (VA)	Fair treatment (VF)
KEEPERS (involved in AVI)	Satisfactory and safety working conditions; professional realization (KW)	Professional freedom (KA)	Fair treatment (KF)
MANAGEMENT STAFF	Satisfactory working conditions; professional realization (MW)	Managerial freedom (MA)	Fair treatment (MF)
VETERINARY STAFF	Satisfactory working conditions; professional realization (VSW)	Professional freedom (VSA)	Fair treatment (VSF)
ZOO	Economic sustainability, support from society (ZW)	Mission fulfillment (ZA)	Adequate legislation and access to resources (ZF)

**Table 3:** Values of risk characterizations for existing control measure and additional control measure to be implemented.

EXPOSURE CONDITION/SCENARIO	HAZARD	CONSEQUENCES	RISK CHARACTERIZATION Phase 4 (Existing Control Measure)				RISK CHARACTERIZATION Phase 5 (Additional Control Measure to be Implemented)			
			P	D	R	RR	P	D	R	RR
Aerosol/Contact with body fluids	*Mycobacterium tuberculosis*, *M. bovis*	Pulmonarytuberculosis/extrapulmonary tuberculosis	1	3	3	L	1	3	3	L
Contact with body fluids	*Brucella* spp.	Fever, arthromyalgia, possible complication and chronicity	1	2	2	L	1	2	2	L
Ingestion: Hand to mouth	*Salmonella* spp.	Mild/severe diarrhea	2	2	4	M	1	2	2	L
Ingestion: Hand to mouth	*Escherichia coli*	Mild/severe diarrhea; Haemolyticuraemic syndrome	2	3	6	M	1	3	3	L
Ingestion: Hand to mouth	*C. jejuni*	Bacteraemia, Guillain-Barre syndrome	2	3	6	M	1	3	3	L
Ingestion: Hand to mouth	*Cryptosporidium* spp.; *C.muris*	Mild/severe diarrhea	2	2	4	M	1	2	2	L
Direct contact	Dermatophytosis	Ring-shaped rash that is reddish and may be itchy	1	2	2	L	1	2	2	L
Foodborn, direct contact (very rare)	*Listeria monocytogenes*	Febrile gastroenteritis, abortion, meningitis, encephalitis, severe septicemia	1	3	3	L	1	3	3	L
Ingestion: Hand to mouth	*Yersinaia enterocolitica*	acute enterocolitis, diarrhea, septicemia	*2*	*2*	4	M	1	2	2	L
Aerosol, contact with infected material and body fluids, ingestion	MRSA, ESBL	Skin infections, UTIs, intra-abdominal and respiratory infections	*2*	*3*	6	M	1	3	3	L
Parts of the body in the projection area of voluntary or involuntary movements of the animal’s neck	Neck blow	Injuries/bone fractures	1	3	3	L	1	3	3	L
Contact with the giraffe’s fur	Urticaria syndrome	erythematosus syndrome/wheals	1	1	1	L	1	1	1	L

P = probability; D = damage; R = risk score; RR = risk rating.

**Table 4:** Frequency of mention of the categories and examples of the reasons given by the respondents (*N* = 55) why the AVI added value to their day at the zoo (in order of frequency).

Categories	Examples	Frequency of Mention N (%) ^1^
Experience/emotions	“They made me feel happy”; “Unique experience”	26 (38.8)
Contact/proximity to animals	“It was great to touch them”; “I have never gotten so close to these animals before”	14 (20.9)
Learning/interest	“I received information that I did not know before”; “It is wonderful to know these animals”	10 (14.9)
Enjoyment	“I enjoyed the experience of giving food to the giraffe a lot”; “Beautiful and funny experience”	5 (7.5)

^1^ Not all respondents completed this survey question.

**Table 5:** Characterization of attributes based on the Kano Model.

Attribute	N (%)					
	M ^1^	L ^2^	E ^3^	I ^4^	R ^5^	Q ^6^
Direct Contact	0 (0)	32 (49)	27 (42)	1 (2)	0 (0)	5 (8)
Providing information about specimens involved	12 (18)	32 (49)	14 (22)	1 (2)	0 (0)	6 (9)
Providing information about conservation issues	5 (8)	44 (69)	13 (20)	1 (2)	0 (0)	1 (2)
Providing information about animal welfare during the interaction	5 (8)	50 (78)	7 (11)	0 (0)	1 (2)	1 (2)

^1^ M=Must-have; ^2^ L=Linear; ^3^ E=Exciter; ^4^ I=Indifferent; ^5^ R=Reverse; ^6^ Q=Questionable response.

## Supplementary Materials

The following are available online at https://www.mdpi.com/2076-2615/9/8/487/s1, Supplemantary 1: preQ questionnare, Supplemantary 2: postQ questionnare, Supplemantary 3: genQ questionnare, Table S1: data visitor experience survey.

## Appendix A

**Table A1:** Details of the multi-facetted animal welfare assessment used in the pilot study (step A–C1).

Method	Component	Description
Behavioral observations and analyses (step A)	Animals and housing	Four male giraffes (*Giraffa camelopardalis*), aged 7 to 15 years, born in captivity, two of them taking an active part in the interactions. Enclosure divided into night enclosure (two straw bedded indoor stalls and a 260 m^2^ outdoor area) and an adjacent naturalistic day exhibit (3600 m^2^ approximately), divided by a ±2.5 m high fence with a gate.
	Observations schedule	For seven interaction episodes recordings took place: For 20 min (five minutes for animal, randomized order) 50 to 30 min before interaction began (PRE-sessions); During the interaction (2 min for animal, several times, same order than in the corresponding PRE-session, DURING-session); For 20 min after the interaction (five minutes for animal, same order than in the corresponding PRE-session, beginning 20–30 min after interaction ended—POST-session). For seven control episodes (similar environmental conditions), recordings were done with analogous time scheduling as that for the interaction episodes (PRE-, DURING-, POST-sessions).
	Data gathering methods	Continuous focal animal sampling, on the video-recordings, using an ad hoc ethogram. Ancillary information gathered on place and modalities of interactions (including the possibility of choice for the animals).
	Variables and statistical analyses	The relative duration of behavior was calculated on the total time each animal was visible in the video-recordings of each session. U Mann-Whitney tests used to compare analogous sessions of the interaction vs. control episodes. Alpha set as 0.05.
Welfare Risk Assessment (step C1)	Method	Semi-quantitative methods were used: The evaluations were carried out in qualitative terms and subsequently transformed into numbers to be processed in calculation algorithms
	Phases	Identification of risk questions, target population, factors of animal welfare concern (checklist), exposure scenarios, known animal welfare consequences and their measurement; Building conceptual models: Scenarios 1, 2, 3 (one scenario for negative subjective experiences, a similar one for injuries, and a different one for infectious diseases transmission from visitors to animals); Proper welfare risk assessment: exposure assessment (frequency of exposure), consequence characterization (assessing the likelihood of consequences and their duration, selecting animal-based indicators of welfare consequences), and risk characterization; Calculating a welfare risk assessment score to be compared to a specifically designed scale, identifying eventually required actions.

**Table A2:** Checklist for risk assessment related to visitors and keepers.

Management Checklist: Preventive and Protective Measures	YES	NO
**1. General preventive security measures**	□	□
1.1. While in the entrance transition zone, did the keeper give adequate information on risks caused by animal contact and on how to reduce them?	□	□
1.2. Did the keeper inform visitors on the possibility to store their personal items in the safe area (where visitors can store their personal items to prevent them from becoming contaminated)?	□	□
1.3. Did the keeper monitor animal behavior during the animal–visitor interaction?	□	□
1.4. Did the keeper monitor visitors’ behavior during the animal–visitor interaction?	□	□
1.5. Did the keeper quit any work activity in case a serious uncontrolled risk is identified (suspect of disease or signs of irritability or aggression)?	□	□
1.6. Did the keeper receive continuous training about biosecurity practices, zoonotic risk, and appropriate practices to minimize these risks?	□	□
1.7. Did the keeper have the knowledge of how to report exposures, accidents, or injuries?	□	□
1.8. Did the keeper have continuous training about the procedures to avoid animal escape?	□	□
1.9. Did the keeper have continuous training to recognize signs of health problems and stress in the animals held in the zoos?	□	□
1.10. Did the keeper check that the animals are free of lesions/illness and/or disease before and after each animal–visitor interaction?	□	□
1.11. Were unauthorized accesses prevented?	□	□
1.12. Were there adequate signs displaying visitors’ rules during interaction (not to smoke, eat, drink, proper hand washing, etc.)?	□	□
1.13. Were there limits to the number of visitors participating in the AVI for each activity?	□	□
1.14. Had an appropriate keepers/visitors/animals ratio been defined?	□	□
1.15. Is there a restriction about access of children under the age of 5 and mentally impaired visitors without supervisor?	□	□
1.16. Was a protocol to avoid animal escaping defined?	□	□
**2. General control measures of zoonotic risk: Biosecurity**	□	□
2.1. Does a site-specific biosecurity plan exist?	□	□
**3. General control measures of zoonotic risk: Veterinary control**	□	□
3.1. Are the veterinarians involved in the management decisions about the species and the individuals who participate in the AVIs?	□	□
3.2. Did the veterinarians compile and follow a preventive, curative, and nutritional veterinary program for the animals?	□	□
3.3. Did the veterinarians perform zoonotic risk analyses?	□	□
3.4. Did the veterinarians update the clinical and pathological records?	□	□
**4. General control measures of zoonotic risk: Environmental hygiene**	□	□
4.1. Did the keeper maintain suitable standards of hygiene to minimize the risk of disease transmission?	□	□
4.2. Were specific protocols or guidelines about the procedures of sanitization?	□	□
4.3. Were the visitor walk-ways cleaned daily?	□	□
4.4. Were the visitor walk-ways cleaned whenever visibly contaminated?	□	□
**5. General control measures of zoonotic risk: Design of the exhibition areas**	□	□
5.1. Was there a dedicate visitor entrance and exit with transition zones?	□	□
5.2. Did the visitors follow a one way flow?	□	□
5.3. Was an animal interaction area, such as an animal enclosure where visitors can touch the animals, clearly defined?	□	□
5.4. Did a service access point exist to differentiate from visitors’ entrance or exit?	□	□
5.5. Does a safe area exist (area where visitors can storage their personal items to prevent them from becoming contaminated)?	□	□
5.6. Were there additional barriers where the visitors enter into the enclosure (to avoid the escape of animals when the visitors enter)?	□	□
5.7. Were there physical safety barriers between the visitors and the animals during the interaction?	□	□
5.8. Was the area of the enclosure where the AVIs occurred well-ventilated?	□	□
5.9. Was the enclosure designed to allow correct cleaning and disinfection?	□	□
5.10. Were visitor walk-ways through the interaction designed to allow for effective cleaning and disinfection?	□	□
5.11. Are an appropriate number of hand-washing stations accessible to all visitors regardless of age or height provided?	□	□
**6. General control measures of zoonotic risk: Control measures for the risks of infection**	□	□
6.1. Did the keeper inform visitors about the rules to be followed during the animal–visitor interaction (e.g., how to touch the animals, not to smoke, drink and eat, act slowly, not to yell, etc.)?	□	□
6.2. Did the keeper recommend the visitor to wash their hands after animal–visitor interaction?	□	□
6.3. Were hand-washing stations, wipes, or antimicrobial gels available after accessing the interaction area?	□	□
6.4. Did automatic (or foot-operated) washing stations have a sufficient water flow volume? Were there an adequate number of soap dispensers, paper towel dispensers, and trash bins?	□	□
6.5. Did the keeper inform visitors about non-edible nature of the food (if the products are provided to visitors to feed the animals)?	□	□
**7. Protective measures: Personal Protective Equipment (PPE)**	□	□
7.1. Were PPE aiming to reduce any risk of contamination to visitors provided?	□	□
7.2. Were PPE aiming to reduce any risk of injuries to visitors provided?	□	□

**Table A3:** Risk characterization (risk score).

Exposure Assessment (D)		Risk = (P × D)			
**Death**	**4**	4	8	12	16
**Serious**	**3**	3	6	9	12
**Mild**	**2**	2	4	6	8
**Lower**	**1**	1	2	3	4
		**1**	**2**	**3**	**4**
		**Rare**	**Unlikely**	**Probable**	**Very likely**
		**Hazard Characterization (P)**			

**Table A4:** Risk categories (risk rating).

Type of Risk	R Value	Action Required
Low risk (L)	R < 4	No action required
Moderate risk (M)	4 ≤ R < 9	Medium-term action (within 1 year)
High risk (H)	9 ≤ R ≤ 12	Urgent action (within 3 months)
Very high risk (V)	R > 12	Immediate action

**Table A5:** Preliminary visitor experience analysis checklist.

Preview Visitor Experience Analysis Checklist on Educational Aspects
1. Did the staff give information about the specimens that are involved in the interaction? □ Yes □ No If yes, please describe. ……………………………………………………………………………………………..
2. Did the staff give information about the biology of the species involved in the interaction activity? □ Yes □ No If yes, please describe ………………………………………………………………………………………………
3. Did the staff give information about animal welfare ^1^? □ Yes □ No If yes, please describe ………………………………………………………………………………………………
4. Did the staff give information about animal welfare issues for the species involved in the interaction activity? □ Yes □ No If yes, please describe ………………………………………………………………………………………………
5. Did the staff give information about wildlife conservation during the interaction activity? □ Yes □ No If yes, please describe ………………………………………………………………………………………………
6. Did the staff suggest behaviors to promote a more suitable lifestyle in order to promote wildlife conservation? □ Yes □ No If yes, please describe ………………………………………………………………………………………………
7. Did the staff suggest behaviors to promote a suitable lifestyle to promote animal welfare? □ Yes □ No If yes, please describe ………………………………………………………………………………………………

**Table A6:** Topics in the visitor experience survey among the three versions.

Topic of the Questions	PreQ	PostQ	GenQ
Demographical variables		×	×
Word association with “giraffa” [giraffe]	×	×	×
Reason to join an animal–visitor interaction (open question)	×		
The expectations of interaction activity (open question)	×		
Recommendation likelihood of the animal–visitor interaction experience—Net Promoter Score (represented by 1–10 scale value)		×	
The perceived value of the experience (represented by Yes/No values with the opportunity to explain)		×	
Pre-information experience (represented by Yes/No values with the opportunity to explain)		×	
Talks already joined in the same day (closed question)		×	×
Animal–visitor interaction already joined in the same day (closed question)			×
Reason not to participate in an animal–visitor interaction (open question)			×
With whom they came to the zoo (closed question with “other” option)		×	×

**Table A7:** List of relevant zoonosis in giraffe and in other animals reported in zoo human–animal interactions included in Phase 1.

Disease	Species	Bibliography
Tuberculosis	*Mycobacterium tuberculosis*; *M. bovis*	[34,35,36]
Brucellosis	*Brucella* spp.	[37,38,39]
Salmonellosis	*Salmonella enterica*	[40,41]
*E. coli* infections	*Escherichia coli*	[42,43,44,45]
Campylobacteriosis	*Campylobacter* spp.	[46]
Cryptosporidiosis	*Cryptosporidium muris*	[47,48]
Listeriosis	*Listeria monocytogenes*	[49]
Yersiniosis	*Yersinaia enterocolitica*	[49]
Antimicrobial-Resistant bacteria	MRSA (methicillin-resistant *Staphylococcus aureus*), ESBL (Extended-spectrum beta-lactamases)	[45,50,51,52]
Dermatophytosis	*Trichophyton* spp.	[53]

**Table A8:** Demographic information for respondents of preQ/postQ and genQ.

Demographic	Category	preQ/postQ Respondents		genQ Respondents		X²	*p*
		Percentage	N	Percentage	N		
Sex	Female	60%	40	63%	67	0.214	0.644
	Male	40%	27	37%	39		
Age	18–24	22%	15	10%	11	9.056	0.107
	25–34	18%	12	30%	32		
	35–44	42%	28	33%	35		
	45–54	15%	10	20%	21		
	55–64	3%	2	6%	6		
	65+	0%	0	1%	1		
Education	Middle school	7%	5	21%	22	6.988	0.072
	High School Graduate	55%	37	44%	47		
	University degree	27%	18	31%	33		
	Other	7%	5	4%	4		
Company ^1^	Friends	9%	6	24%	25	5.783	0.016
	Consort	70%	47	65%	69	0.693	0.405
	Son/s	51%	34	54%	57	0.151	0.698
	Others	16%	11	12%	13	0.657	0.418
Annual ticket/Membership	Yes	0%	0	9%	10	6.611	0.01
	No	99%	66	91%	96		
Number of past visits	First time	82%	55	52%	55	17.45	<0.001
	More than once	16%	11	48%	51		
Natural childhood	During all the year	54%	36	43%	46	3.923	0.27
	During the summer	27%	18	31%	33		
	Rarely	15%	10	24%	25		
	Other	0%	0	2%	2		
Pet ownership	Have a pet	67%	45	64%	68	0.68	0.41
	Not have a pet	28%	19	36%	38		

^1^ For this item, multiple answers were allowed.

**Table A9:** Frequency of mention of the categories and examples of individual motivation identified when respondents (*N* = 67) were asked why they decided to join the “giraffe feeding” interaction (in order of frequency).

Categories	Examples	Frequency of Mention N (%) ^1^
Contact/proximity to animals	“I love interacting with animals”; “Opportunity to get closer”	29 (21.5)
Appreciation for animals	“Because I like animals”; “Because they are beautiful animals”	27 (20)
Learning/interest	“To know it better”; “Because I am interested in their behavior”	19 (14)
Experiences/emotion	“To test myself”; “I have never fed a giraffe before”	18 (13.3)
Because of the children	“To let my children live a unique experience”; “To share a special moment with my little baby”	18 (13.3)
Curiosity	“Curious about their life”; “Curiosity”	17 (12.6)
Other	“It was the only interaction available”; “Entertainment”	7 (5.2)

^1^ Not all respondents completed this survey question.

**Table A10:** Frequency of mention of the categories and examples of individual motivation given when respondents (*N* = 79) who had not joined any animal–visitor interaction on the day of the survey were asked the reason (in order of frequency).

Categories	Examples	Frequency of Mention N (%) ^1^
Not possible to schedule today	“We were late”; “We did not have enough time”	20 (25.3)
Not interested	“I did not think about it”; “We have done something else”	15 (19)
Not willing to pay	“Too expensive”; “I did not want to pay for extra experiences”	11 (13.9)
Do not know about them	“I did not know the timetable”; I did not know it was possible to join them”	9 (11.4)
Prefer to relax/Pool	“Too hot, we went to the pool”; “Pool”	7 (8.9)
Done in the past/Already scheduled for the future	“I already join them in the past”; “Tomorrow”	6 (7.6)
Children are too small	“Two small kids to manage”; I was with my daughter, she is too small”	4 (5.1)
Do not like	“Distrust with animals I do not know”; “Also inside the enclosure? No, poor animals”	4 (5.1)

^1^ Not all respondents completed this survey question.

**Table A11:** Customized ethical matrix.

Respect for	WELLBEING	AUTONOMY	FAIRNESS
ZOO GIRAFFES PARTICIPATING IN THE AVI	Improving animal health and avoiding risks to animal welfare; ability to express normal patterns of behavior; being kept in an appropriate space, with an adequate number of individuals; living a life worth living for a wild animal in human care. (ZGW)	Behavioral freedom: ability to choose not to interact with visitors; being able to have a degree of control on the environment. (ZGA)	Being considered with respect as “ambassador animals” contributing to the conservation of the species in the wild. Same opportunities for positive welfare outcomes as other animals that are not involved. (ZGF)
ZOO GIRAFFES NOT PARTICIPATING IN THE AVI	Improving animal health and avoiding risks to animal welfare; ability to express normal patterns of behavior; being kept in an appropriate space, with an adequate number of individuals; living a life worth living for a wild animal in human care. (GW)	Behavioral freedom: ability to choose to interact with visitors; being able to have a degree of control on the environment; (GA)	Being considered with respect as “ambassador animals”, contributing to the protection of the species in the wild. Same opportunities for positive welfare outcomes as other animals involved in the interactions. (GF)
WILD GIRAFFES AND THE ENVIRONMENT	To have an adequate environment in which to live, without natural and/or human threats that endanger their conservation; living a life worth living in the natural habitat. (WW)	Having the freedom to choose where to live and to reproduce; having the availability of sufficient resources. (WA)	Living without threats caused by man—directly or indirectly; having the right to a legal protection that safeguards them in space and time; being part of conservation and reintroduction projects; to have ambassadors of their species in zoos who are viewed with respect. (WF)
VISITORS PARTICIPATING IN THE AVI	See exotic and wild animals and learning information and be educated about them. Safety and secure environment and amusement. (AW)	See the animals; have access to information on animals, on environments and on conservation; possibility to do educational and entertainment activities; take part in projects of conservation of nature. (AA)	Affordability; be physically and emotionally close to the nature and animals; spent pleasantly days and do emotional and educational activities. (AF)
VISITORS NOT PARTICIPATING IN THE AVI	Learning information and being educated about wild animals if interested in; safety and secure environment; support the zoo mission statement and activities. (VW)	Having access to all the information about the activities; having the freedom to choose whether to participate in the activity or not; having the opportunities to see the animals; having access to the information on animals, conservation and environments; being free to take part in conservation projects. (VA)	Equal educational opportunities and equal opportunities of accessing the natural resources; equal right to being physically and emotionally close to nature and animals; equal right to benefit from the mission statement of the zoo (in terms of welfare, conservation, and education). (VF)
KEEPERS INVOLVED IN THE AVI	Having an economically rewarding and comfortable job; working in a safe and secure environment, also during the interactions. (KW)	Being able to choose the tasks that best reflect personal skills and values; being able to be part of the management strategies to promote the well-being of the animals involved in the interactions; being able to be part of the management strategies to promote the conservation and education activities related to interactions; being able to work independently (in terms of space, instruments, skills, and education) and respecting the law; to be always updated on the new research about animal welfare, conservation, education and the current legislation. Being able to participate in scientific projects and for the protection of nature as a whole. (KA)	Respect for their role; equal right to professional practice; equal access to funds to develop and grow; be able to have a clear and adequate legislation that protect at the working place; contributing to the fulfilling of the mission statement of the zoo, in terms of welfare, conservation and education. (KF)
MANAGEMENT STAFF	Having an economically rewarding, comfortable job; working in a safe and secure environment; having the professional support of an expert/qualified/trained staff. (MW)	Being able to choose the best strategies to promote the well-being and conservation of individuals, groups, and species; being able to choose the best strategies to promote the education activities; being able to work independently (in terms of space, instruments, skills, and education) and respecting the law; to be always updated on the new research about animal welfare, conservation, education the current legislation. (MA)	Equal right to professional practice; equal access to funds to develop and grow; be able to have a clear and adequate legislation that protect at the working place; being recognized in their role in the fulfilling of the mission statement of the zoo, in terms of welfare, conservation and education. (MF)
VETERINARY STAFF	Having an economically rewarding and comfortable job; working in a safe and secure environment; having the opportunity to guarantee the physiological and psychological welfare of the animals housed in the zoo; having the opportunity to self-realization and personal fulfillment; (VSW)	Being able to work independently (spaces, instruments, skills, and education) and in total respect of the laws; being able to participate in scientific projects and for the protection of nature as a whole; have the possibility of being able to fulfil the ethical code of the profession; being able to contribute to choose the best strategies to promote and maintain the well-being of individuals, groups, and species; to be able to take decisions about the health of the animals and feasibility of interactions when animals’ health and welfare issues are concerned; having the necessary resources to prevent and treat diseases, to eliminate or reduce pain, suffering, injury, and fear and to promote well-being of the animals involved. (VSA)	Respect for their job and professional skills; being recognized as an advocate for the well-being of the animals, especially the ones that are involved in the interactions. (VSF)
ZOO	Having the support and the approval of society and Institutions in order to carry out conservation projects; having access to funds and to a satisfactory income; guarantee a safe environment for the visitors, the staff and the animals; guarantee educational and enjoyable activities for the visitors. (ZW)	Being able to carry out their mission (education, conservation, research) and to maintain high standards of wellbeing of the hosted animals; being able to collaborate with all the stakeholders in order to be consistent and to adopt a transparent regulation; to be able to follow European guidelines that guarantee the homogeneity of actions and norms; be able to be in contact with other institutions and facilities in order to be always updated. (ZA)	Having a mission statement and being able to fulfil it; to have the funds to develop, and grow; being able to have a clear and adequate legislation and indications on the rules to be respected; having consistent, up-to-date and effective internal legislation and competent inspections by the authorities in order to guarantee the standards required; promoting equal educational and entertainment opportunities. (ZF)
