# Supplementary material for: A Protocol for the Ethical Assessment of Wild Animal–Visitor Interactions (AVIP) Evaluating Animal Welfare, Education, and Conservation Outcomes
Source: Animals (Basel). 2019 Jul 25;9(8):487. doi: 10.3390/ani9080487 (PMC6721246; doi:10.3390/ani9080487)
Supplement: Supplementary file 1 [file animals-09-00487-s001.zip › supplementary/genQ_questionnaire.pdf]

DIPARTIMENTO DI BIOMEDICINA COMPARATA E ALIMENTAZIONE

ZOOM

To be filled in by the operator GenQ	☐ **pool** ☐ **park**
date……………………………………/ hour ……………………………/ operator …………………………………………	

Privacy statement

1. Age (years old) ……………… 2. Sex ☐ M ☐ F	3. Educational level<br>☐ Middle school<br>☐ High school Graduate<br>☐ University degree<br>☐ Other<br>……………………………………………………………

4. Write the first three words that come to your mind when you think of a turtle.

____________________ ____________________ ____________________

5. Write the first three words that come to your mind when you think of a giraffe.

____________________ ____________________ ____________________

Some information about your day at ZOOM

6. Have you joined any of the interactions offered by the park (e.g. interaction with tortoises, giraffes or birds of prey called Extra Experience)?
(Please note that the interactions are the experiences that need an extra ticket and allowed you to enter in the enclosure with the animals) (*Mark all that apply)*

☐ yes, I participated in *"Giraffe feeding"*
☐ yes, I participated in *"A tu per tu con le tartarughe"*
☐ yes, I participated in *"Diventa Falconiere"*
☐ no, I did not participate in any of these activities

7. If you answered NO in the previous question, why did you not participate in these activities? *(If you answered YES, skip this question)*

……

8. Have you already joined any of these talks with biologists/keepers today? (Mark all that apply)

☐ "Conosci le tartarughe"
☐ "Gli animali del Serengeti"
☐ Other talks not listened here
☐ None

9. With whom are you at the zoo today? *(Mark all that apply)*

☐ with friend/s
☐ with my husband/wife /partner
☐ with my child/children
☐ other (*specify*) ………………………………

10. How many times have you already visited ZOOM?

☐ first time
☐ more than once

11. Do you own the ZOOM's annual ticket?

☐ yes ☐ no

Some information about you

12. Did you spend days in contact with nature during your childhood?

☐ Yes, during all the year
☐ Yes, during the summer
☐ No, rarely
☐ other (*specify*) …………………………………………..

13. Do you own pets at home? *(Mark all that apply)*

☐ no, I do not have any pet
☐ yes, a dog/s
☐ yes, a cat/s
☐ yes, a fish/fishes
☐ yes, an hamster/s
☐ yes, a bird/s
☐ yes, a turtle/s
☐ yes, a snake/s
☐ other (*specify*) ………………………

Leave us your email adress if you would like to be involved in future conservation projects.

……………………………………………………………………………………………………..

Thank you for your contribution!
