# Supplementary material for: A Protocol for the Ethical Assessment of Wild Animal–Visitor Interactions (AVIP) Evaluating Animal Welfare, Education, and Conservation Outcomes
Source: Animals (Basel). 2019 Jul 25;9(8):487. doi: 10.3390/ani9080487 (PMC6721246; doi:10.3390/ani9080487)
Supplement: Supplementary file 1 [file animals-09-00487-s001.zip › supplementary/postQ_questionnaire.pdf]

DIPARTIMENTO DI BIOMEDICINA COMPARATA E ALIMENTAZIONE

ZOOM

To be filled in by the operator	PostQ	operator ....

(Privacy statement here)

1. Age (years old)<br>.................<br>2. Sex<br>☐ M ☐ F	3. Educational level<br>☐ Middle school<br>☐ High school Graduate<br>☐ University degree<br>☐ Other<br>..

4. Write the first three words that come to your mind when you think of a giraffe.

____________________ ____________________ ____________________

5. Would you suggest to a friend to participate in the experience? Mark with an *X* on a scale from 1 to 10, where 1 means "absolutely not", and 10 "absolutely yes".

1 2 3 4 5 6 7 8 9 10

6. Do you think that the experience with the giraffes added value to your day at Zoom?

☐ yes, because...

☐ no

7. Are you satisfied with the information provided by the staff before the experience?

☐ yes, because ....

☐ no, because ....

Some information about your day at ZOOM

8. Which of these talks with biologists/keepers have you already joined today? *(Mark all that apply)*

☐ "Conosci le tartarughe"
☐ "Gli animali del Serengeti"
☐ Other talks not listed here
☐ None

9. With whom are you at the zoo today? *(Mark all that apply)*

☐ with friend/s
☐ with my husband/wife/partner
☐ with my child/children
☐ other (*specify*) ………………………………

10. How many times have you already visited ZOOM?

☐ first time
☐ more than once

11. Do you own ZOOM's annual ticket?

☐ yes ☐ no

Your opinions and impressions

12. Mark with an *X* the option that best describes how you feel about each of these questions on the "Giraffe feeding" experience.

1a) If during the interaction, you can touch a giraffe, how do you feel?	☐ Like
	☐ Must
	☐ Do not care
	☐ Can live with it
	☐ Dislike
1b) If during the interaction, you can **not** touch any giraffe, how do you feel?	☐ Like
	☐ Must
	☐ Do not care
	☐ Can live with it
	☐ Dislike
2a) If during the interaction, you are told the age, sex and particular characteristics of the giraffe you interact with, how do you feel?	☐ Like
	☐ Must
	☐ Do not care
	☐ Can live with it
	☐ Dislike
2b) If during the interaction, you are **not** told the age, sex and particular characteristics of the giraffe you interact with, how do you feel?	☐ Like
	☐ Must
	☐ Do not care
	☐ Can live with it
	☐ Dislike

3a) If during the interaction, you are suggested actions you can do to help the conservation of the species you are interacting with, how do you feel?	☐ Like<br>☐ Must<br>☐ Do not care<br>☐ Can live with it<br>☐ Dislike
3b) If during the interaction, you are **not** suggested any action you can do to help the conservation of the species you are interacting with, how do you feel?	☐ Like<br>☐ Must<br>☐ Do not care<br>☐ Can live with it<br>☐ Dislike
4a) If during the interaction, you are suggested behaviours you can do to preserve the animal welfare during the interaction, how do you feel?	☐ Like<br>☐ Must<br>☐ Do not care<br>☐ Can live with it<br>☐ Dislike
4b) If during the interaction, you are **not** suggested any behaviour you can implement to preserve the animal welfare during the interaction, how do you feel?	☐ Like<br>☐ Must<br>☐ Do not care<br>☐ Can live with it<br>☐ Dislike

Some information about you

13. Did you spend days in contact with nature during your childhood?

☐ Yes, during all the year
☐ Yes, during the summer
☐ No, rarely
☐ other (*specify*) ………………………………………..

14. Do you own pets? *(Mark all that apply)*

☐ no, I do not have any pet
☐ yes, a dog/s
☐ yes, a cat/s
☐ yes, a fish/fishes
☐ yes, an hamster/s
☐ yes, a bird/s
☐ yes, a turtle/s
☐ yes, a snake/s
☐ other (*specify*) ………………………

Leave us your email adress if you would like to be involved in future conservation projects.

………………………………………………………………………………………………………..

Thank you for your contribution!
