# Supplementary material for: A Protocol for the Ethical Assessment of Wild Animal–Visitor Interactions (AVIP) Evaluating Animal Welfare, Education, and Conservation Outcomes
Source: Animals (Basel). 2019 Jul 25;9(8):487. doi: 10.3390/ani9080487 (PMC6721246; doi:10.3390/ani9080487)
Supplement: Supplementary file 1 [file animals-09-00487-s001.zip › supplementary/preQ_questionnaire.pdf]

DIPARTIMENTO DI BIOMEDICINA COMPARATA E ALIMENTAZIONE

ZOOM

To be filled in by the operator PreQ operator………………………………………
Date……………………………………hour ……………………………/ questionnaire n. ………………………

(Privacy statement here)

1. Write the first three words that come to your mind when you think of a giraffe.

______________________ ______________________ ______________________

2. Why have you decided to join this activity with giraffes?

a) ……………………………………………………………………………………………
b) ……………………………………………………………………………………………
c) ……………………………………………………………………………………………

3. Which are your expectations about this activity?

a) ……………………………………………………………………………………………
b) ……………………………………………………………………………………………
c) ……………………………………………………………………………………………

Thank you for your contribution!
